# Exploiting Trap Type and Color for Monitoring Macadamia Felted Coccid *Acanthococcus ironsidei* (Williams) and Associated Parasitic Wasps in Macadamia Orchards in Hawai’i

**DOI:** 10.3390/insects16020149

**Published:** 2025-02-02

**Authors:** Angelita L. Acebes-Doria, Pascal O. Aigbedion-Atalor

**Affiliations:** 1 Daniel K. Inouye US Pacific Basin Agricultural Research Center, United States Department of Agriculture, Agricultural Research Service, 64 Nowelo St., Hilo, HI 96720, USA; 2 Oak Ridge Institute for Science and Education, Oak Ridge Associated Universities, Oak Ridge, TN 37830, USA; atalor@hawaii.edu; 3 Department of Plant and Environmental Protection Sciences, College of Tropical Agriculture and Human Resilience, University of Hawaii at Manoa, 3050 Maile Way, Gilmore 513, Honolulu, HI 96822, USA

**Keywords:** tree nut, invasive pest, Hemiptera, trap enhancement, natural enemies, *Encarsia*, biological control, Hymenoptera

## Abstract

*Acanthococcus ironsidei* (Williams) (Hemiptera: Eriococcidae) is an invasive pest of macadamia, *Macadamia integrifolia*, in Hawai’i, causing death to macadamia trees and decreased nut productivity. Monitoring relies on wrapping double-sided sticky tapes over tree branches to trap dispersing crawlers (i.e., mobile immature stage), but this is tedious for growers, especially in large orchards. From September to November 2022 and December 2022 to February 2023, at two commercial macadamia orchards on Hawai’i Island, the use of colored sticky cards was assessed for improving the monitoring of *A. ironsidei* and to investigate the Hymenopteran parasitoid complex that inhabits macadamia canopies. At each study site, four different colored sticky cards (yellow, lime green, dark green, and white) were placed on the lower canopy of five trees, and on each tree, a transparent double-sided sticky tape was deployed. At bi-weekly intervals, the sticky cards were replaced and re-randomized on each tree, and the double-sided sticky tapes were replaced. The results showed that the sticky cards captured both *A. ironsidei* crawlers and (winged) male adults, while the double-sided sticky tapes captured only crawlers. The trap color did not have significant effects on the captures of *A. ironsidei* male adults at the sites, while the captures of crawlers on sticky cards were lowest on the dark green sticky traps at one site. The captures of *A. ironsidei* adult males on white sticky traps were generally correlated with the number of crawlers captured on the double-sided sticky tapes. The parasitoid complex captured had disparities in the attraction to color; however, the yellow, lime green and dark green colors were seemingly more effective for monitoring *Encarsia lounsburyi* (Berlese & Paoli), a reported parasitoid of *A. ironsidei*. These results have useful practical implications for improved monitoring of *A. ironsidei* crawlers, male adults and associated natural enemies.

## Simple Summary

The invasive insect pest *Acanthococcus ironsidei* (Williams) (Hemiptera: Eriococcidae) is a serious pest of macadamia, *Macadamia integrifolia*, in Hawai’i. It feeds on all parts of the macadamia shoot and can cause death to macadamia trees and losses in nut production, especially when infestation levels are high. The current method for monitoring the densities of the pest requires wrapping tree branches with double-sided sticky tapes. However, this monitoring method is laborious and usually not feasible in large orchards. This study aimed to assess whether a different trap type and color would improve the trapping efficiency in terms of the pest and its associated hymenopteran parasitoids in large orchards. To accomplish this, dark green, lime green, white, and yellow sticky cards, each ~1 m apart, were placed on five macadamia trees (=replicates), each with a double-sided sticky tape, at two commercial macadamia orchards in Hawai’i for six months (September–November 2022 and December–February 2023). The sticky cards were collected and replaced every two weeks, moving each replicate to a new position on the tree. The numbers of *A. ironsidei* immature crawlers, male adults, and hymenopteran parasitoids captured on the sticky cards and double-sided sticky tapes were recorded. Overall, the sticky cards improved the efficiency of monitoring *A. ironsidei* by capturing crawlers and male adults compared to the double-sided tape, which captured only crawlers. However, the trap color was not influential in attracting male adults. A positive correlation was found between the male adults captured on certain colors of sticky cards and the crawlers captured on the double-sided sticky tapes. *Encarsia lounsburyi* (Berlese & Paoli) was the most abundant parasitoid at both sites, and colored traps, rather than white, were more efficient in trapping this species.

## 1. Introduction

*Macadamia integrifolia* (Maiden & Betche) (Proteaceae) (hereafter “macadamia”) is one of the most valuable crop commodities in Hawai’i, with an estimated farm value of ~USD 53.9 million [1]. Several insect pests attack macadamia in Hawai’i and the macadamia felted coccid, *Acanthococcus ironsidei* (Williams) (Hemiptera: Eriococcidae), native to Australia, is one of the worst. The first documented record of *A. ironsidei* in Hawai’i was an interception of an infested macadamia species imported onto the island in 1954; however, based on the report by Williams [2], it is believed that *A. ironsidei* did not establish at this time [1]. Conversely, surveys conducted in South Kona in late February 2005 confirmed the presence and establishment of *A. ironsidei* in Hawai’i [3]. The results of the initial sampling efforts to understand the invaded range indicated that the pest seemed to be confined to South Kona [3,4]. However, further monitoring showed that *A. ironsidei* had rapidly spread eastward and north of Kona, resulting in severe infestations in many macadamia orchards [4].

Currently, the invaded range of the pest in Hawai’i has increased and its damage to macadamia production and the farm economy is significant [1]. For example, the annual losses per farm attributed to *A. ironsidei* can be over half a million pounds of wet in-shell macadamia nuts [1]. The pest can cause tree damage and death by feeding on all parts of the shoot [5]. *Acanthococcus ironsidei* adopts a modified form of incomplete or hemimetabolous metamorphosis [6,7]. Eggs eclose to the next immature stage, crawlers—forming a felted sac that can cover their bodies, and subsequently develop into either adult females that are sessile or males that are winged [7]. The damaging life stages are the crawlers and female adults, penetrating the epidermis with their thread-like stylet to extract plant fluids [3,7]. High crawler and female infestations distort and stunt new tree growth, causing yellow spotting on older leaves. If left untreated, high infestations can cause dieback, decline in the nut yield, and delay in the fall of mature nuts [3].

After the arrival, establishment and proliferation of invasive pests in new environments, farmers, especially in agricultural and horticultural landscapes, often respond with control measures that rely on insecticides [8,9,10]. Although insecticides are important tools for controlling insect pest populations [11,12,13], over-reliance can be counterproductive due to mechanisms that facilitate the evolution of insecticide resistance in insects [9,12,13,14]. In this context, integrated pest management (IPM)—an approach that can harness and synchronize several multidisciplinary control strategies, resulting in a comprehensive management action plan—is often recommended [15,16,17,18]. Also, growing global concerns about the use of insecticides due to ample evidence showing their inimical effects on environmental and human health consolidate the need for IPM [11,14,19,20,21].

Substantial efforts have been invested in developing effective IPM strategies to control *A. ironsidei* in Hawai’i [1]. Conant et al. [3] highlighted the need to explore some natural enemies, such as predaceous ladybird beetles, predatory moth larvae, parasitic wasps, lacewings, and predatory mites, underscored by Ironside et al. [5] as co-evolved natural enemies that regulate the population of *A. ironsidei* in Australia. Also, Wright and Conant [4] explored the use of horticultural oils—Saf-T-Side^®^ insecticide—and found them effective in suppressing populations of *A. ironsidei* when applied correctly with a hardy air-assisted blower. More recently, the need to develop efficient monitoring tools that can provide relevant information on the infestation levels of *A. ironsidei* to guide decisions encompassing the deployment and application of control measures for timely targeted interventions has been documented. In this context, Gutierrez-Coarite et al. [22] reported that *A. ironsidei* crawlers can be monitored with sticky tapes by wrapping a two-sided sticky tape around a lower branch (1.5–2 m above ground) and an upper branch (3–4 m above ground) of the tree. Also, recently, the use of unmanned aerial vehicles (UAVs), or “drones”, has been assessed as a potential tool for precision monitoring of *A. ironsidei* [23].

The visual cues for orientation in insects encompass the color, shape, size, and certain physical cues associated with host species [24]. Insects have the capacity to discriminate wavelengths of light independent of the intensity [25,26], and there is ample literature highlighting that the pattern and color of sticky traps significantly mediate the performance of the traps [27,28,29]. For example, the Asian citrus psyllid, *Diaphorina citri* Kuwayama (Hemiptera: Liviidae), a vector of the bacteria *Candidatus* Liberibacter—the putative causal agent of the devastating citrus greening disease Huanglongbing—is attracted more to yellow sticky traps than to the blue, green, red, and white counterparts [30]. Also, Rodriguez-Saona et al. [31] showed that leafhopper species have clear preferences for certain colors, differing in intensities along a spectrum of wavelengths in cranberry, *Vaccinium macrocarpon* Ait. bogs. These authors found that green, red, and yellow, in that order, were the most attractive colors for the blunt-nosed leafhopper *Limotettix* (=*Scleroracus*) *vaccinii* (Van Duzee) (Hemiptera: Cicadellidae), while the sharp-nosed leafhopper *Scaphytopius magdalensis* (Provancher) (Hemiptera: Cicadellidae) was attracted more to yellow [31]. More recently, Mahot et al. [32] reported that *Sahlbergella singularis* Hagl. (Hemiptera: Miridae), a major pest of cacao (*Theobroma cocoa* L.), is attracted more to sex-pheromone-baited traps that are colored green than to those colored white and purple. Evidently, hemipterans, like many other insect groups, are influenced by color [32,33,34,35,36]. Trap color testing and optimization have not been conducted for *A. ironsidei* monitoring in Hawai’i. To date, only a transparent double-sided sticky tape has been explored for monitoring *A. ironsidei* and there are no tools for monitoring its potential natural enemies in macadamia orchards. Sticky cards offer a sustainable and effective means of monitoring most soft-bodied hemipterans and associated parasitoids, and color optimization can improve their efficiencies [37]. Therefore, this study aimed to assess if the trap type (sticky tape vs. sticky card) and color could be exploited to improve the efficiency of sticky tapes used for monitoring *A. ironsidei* and parasitoids associated with the pest in macadamia orchards in Hawai’i.

## 2. Methods

This study was conducted at two commercial macadamia orchards with previously known *A. ironsidei* infestation in Paauilo and Pahala on Hawai’i Island. The surveys were administered from September to November 2022 and from December 2022 to February 2023, corresponding to the two distinct periods of *A. ironsidei* population dynamics, as reported by Guttierez-Coarite et al. [22], with the latter being a low-activity period and the former being a high-activity period.

Different colored sticky cards, including yellow, lime green, dark green and white, were compared at each site (Supplementary Figure S1A, inset). Each sticky card (Alpha Scents, Inc., Canby, OR, USA) was standardized at 12.7 cm × 17.78 cm. Five mature macadamia trees with active *A. ironsidei* infestations within a one-hectare block were used for this study. One replicate of each color was hung on the lower canopy of each tree at ~1 m apart, and five replicates of each color were established at each site. A transparent double-sided sticky tape (2.54 cm width, Gorilla Glue, Cincinnati, OH, USA), the current monitoring trap for *A. ironsidei* crawlers, was deployed on one branch of each tree with the different colored sticky cards (Supplementary Figure S1B). Every two weeks, the sticky cards were replaced and re-randomized in each tree, and the double-sided sticky tapes were changed.

The numbers of *A. ironsidei* male adults, crawlers and various parasitic hymenopteran families captured on all the sticky cards and tapes were counted and summarized for each site for each study period. For the identification of captured specimens, appropriate taxonomic references were used [7,38,39,40] and confirmed using voucher specimens identified by taxonomists. For each group of captured insects, a one-way ANOVA using a generalized regression model, with either a zero-inflated Poisson or Poisson distribution, was conducted to examine the differences in the capture rates across the different sticky card colors, followed by Tukey’s post hoc test for multiple mean comparisons. Correlation analyses were also performed to examine the relationship between the adult males of *A. ironsidei* captured on the sticky cards and the *A. ironsidei* crawlers captured on the double-sided sticky tapes. Captures on the double-sided sticky tapes were standardized at 6.4516 cm^2^. All the statistical analyses were conducted using JMP Pro Version 17.0 (SAS Institute Inc., Cary, NC, USA, 1989–2023).

## 3. Results

Relative captures across all traps (Supplementary Tables S1–S4). The sticky cards, in general, captured diverse insects, including adult and immature *A. ironsidei* (=crawlers) and various species of parasitic hymenopterans from different families. Regardless of the location and sampling period, relatively more male *A. ironsidei* adults (>14–71%) than crawlers (0–11%) were captured on the sticky cards, while only crawlers were captured solely on the double-sided sticky tapes (~99%).

Sticky card color comparisons of *A. ironsidei* captures. In Pahala (Table 1 and Table 2), no differences in the captures of *A. ironsidei* adult males and crawlers among the different colored sticky cards were recorded, while in Paauilo (Table 3 and Table 4), the captures of *A. ironsidei* crawlers were consistently highest on the white cards and lowest on the dark green cards, regardless of the trapping period.

Sticky card color comparisons of Hymenopteran captures. Consistently across the two trapping periods in Pahala (Table 1 and Table 2), the captures of *Encarsia* spp., Myrmaridae and Aphelinidae were significantly different among the different trap colors. Yellow cards captured the most *Encarsia* spp. versus the other colors; yellow, lime green and dark green colors captured more Myrmaridae than white cards; while yellow and lime green cards consistently had the highest Aphelinidae captures. The captures of *Signiphora* spp., Trichogrammatidae and other hymenopteran wasps were different across the trap colors during the September to November trapping period (Table 1), but no differences were found from December through February (Table 2). The *Signiphora* spp. and other Hymenopteran captures were highest on yellow cards, while Trichogrammatidae wasps were captured more on dark green cards. The captures of *Encarsia lounsburyi* and Eulophidae wasps were equal across all the trap colors.

In Paauilo (Table 3 and Table 4), the captures of myrmarid wasps were consistently highest on lime green cards and lowest on white cards during the two trapping periods. Differences in the trap captures among the trap colors were only detected from December to February for *Encarsia lounsburyi* and *Signiphora* spp. (Table 4). *Encarsia lounsburyi* wasps were captured equally on yellow, lime and dark green cards in high numbers compared to white cards. The *Signiphora* spp. captures were highest on yellow cards in comparison to the other colors. No differences in the captures of *Encarsia* spp., Trichogrammatidae, Aphelinidae, Eulophidae and other hymenopterans were found among the different trap colors.

Correlations between *A. ironsidei* captures on double-sided sticky tapes vs. different colored sticky cards. The numbers of *A. ironsidei* male adults captured on white sticky cards were positively correlated with the *A. ironsidei* crawlers captured on the double-sided sticky tapes from September to November at both study sites and from December to February in Paauilo (Table 5). The captures of *A. ironsidei* were also correlated between the lime green sticky cards and the double-sided sticky tapes in Pahala from December to February, and between the yellow cards and the double-sided sticky cards in Paauilo from September to November. No correlations were found between the *A. ironsidei* captures on dark green sticky cards and double-sided sticky tapes across the sites and trapping periods.

## 4. Discussion

Early infestations of *A. ironsidei* are frequently unnoticed, mainly because of the pest’s inconspicuous nature and the recurring infeasibilities associated with physically sampling large orchards [1,4,22,23]. The population of *A. ironsidei* can rapidly increase to severe infestations, causing the death of macadamia tree branches and the characteristic copper-colored dead leaves [1,23]. Currently, populations of *A. ironsidei* are quantified across macadamia orchards in Hawai’i by counting the crawler numbers per unit area of the bark of infested trees [22,23]. In this process, a coarse-grit sandpaper is used to smoothen a selected rough bark area on a branch or the main trunk, and a double-sided sticky tape is wrapped around the sanded area, which then captures mobile flightless crawlers for monitoring tree infestations [22,23]. Although this approach is generally considered important for monitoring populations of *A. ironsidei*, recommendations for improvements have also been documented [23]. In this context, the results of this study showed that sticky cards can be used complementarily to double-sided sticky tapes as they increased the overall captures of *A. ironsidei* as well as obtained information on associated parasitoids. The sticky cards were effective in trapping winged male adults of *A. ironsidei*, while the double-sided tapes were effective in trapping dispersing crawlers across both study sites. Male adults and crawlers are the dispersive and mobile stages of *A. ironsidei* as female adults are flightless and sessile. Signs of dead *A. ironsidei* females remain on the leaves and other plant parts even long after an active infestation, and thus, monitoring the number of alive females can be tedious and require a lot more training, especially for growers. Therefore, monitoring the activity of adult males and/or crawlers can be a better alternative for determining active infestation levels in the field. It can be argued that sticky cards can be used in lieu of the double-sided sticky tapes if trapping *A. ironsidei* male adults suffices for monitoring *A. ironsidei* populations for pest management purposes. Deploying sticky cards and processing samples from sticky cards are simpler and less cumbersome than doing so with double-sided sticky tapes, which makes it more efficient and feasible for monitoring *A. ironsidei* than sticky tapes, especially in large orchards. The results from this study are foundational in using sticky cards as a monitoring approach for *A. ironsidei* infestation levels. Thus, a threshold for *A. ironsidei* management using sticky cards should be developed, similar to the established economic injury level thresholds based on double-sided sticky tapes [41].

The results also support the hypothesis that color may be consequential in designing and optimizing traps for monitoring *A. ironsidei* in Hawai’i. Although there were no significant differences in the colored sticky trap captures of male adults across both study sites, the white-colored traps had the most crawler captures, especially when the infestation rates of both crawlers and adult males of *A. ironsidei* were high. On the other hand, dark green was seemingly the least attractive color for trapping crawlers. The implications of these findings somewhat suggest that color may not necessarily be a critical factor in trapping male adults during low or high infestation periods, as all four colors (i.e., yellow, lime green, dark green, and white) did not show any significant differences, but the yellow cards had the highest average captures across both sites. From these results, we deduce the following: optimizing monitoring tools with the (1) addition of white sticky cards for crawlers of *A. ironsidei* with the double-sided sticky stapes, if increased sensitivity to crawler activity is deemed relevant for monitoring purposes, and/or (2) use of yellow sticky cards for monitoring adult male activity in lieu of double-sided tape, if adult activity monitoring is the primary purpose. We do emphasize that additional studies are needed to investigate the economic injury levels based on adult activity monitored using sticky cards, and the costs associated with this approach would also need to be considered.

In agroecosystems, populations of insect pests are regulated mainly by naturally occurring organisms such as predators, parasites, and parasitoids [42,43,44,45,46]. In a system that is out of balance or released from natural enemy regulation, pest populations can explode, with devastating consequences [17,47], as is the case with many invasive pests, including *A. ironsidei* in Hawai’i [1,3,4,22,48,49]. Many predators can consume multiple hosts until satiation, while nutrition for parasites is obtained from individual hosts without killing them—the parasite–host relationship seldom results in host death because the parasite requires the host to be alive to survive [50,51,52,53]. However, parasitoids utilize (feed and/or kill) a single host individual—because the parasitoid–host relationship results in host death [54,55], and many IPM programs often utilize hymenopterans for managing target pest species [42,56]. Understanding the distribution and abundance of these parasitoids in cropping systems such as macadamia is crucial for developing, implementing, and optimizing effective IPM strategies [57,58,59], including an effective monitoring tool such as the use of colored traps [60,61]. Nevertheless, insights into the spatiotemporal fluctuation of target pests are imperative for understanding if their population trends synchronize with the occurrence and abundance of their associated parasitoids [47,62,63,64]. Here, the infestation rates of *A. ironsidei* across both sites showed differential patterns. From September to November, the infestation rates were high at Pahala and declined between December and February. Conversely, the infestation rates of *A. ironsidei* at Paauilo were high during the two sampling periods, with the crawler population exceeding 10,000 between December and February 2023. This finding correlates with other reports showing high infestations of *A. ironsidei* in macadamia orchards in Hawai’i [22]. It also suggests that for effective natural enemy regulation of *A. ironsidei*, the populations of effective natural enemies should exceed thresholds that can exert substantial control pressure on the pest’s population to reduce severe infestations that result in nut losses and tree losses.

Several natural enemies, including hymenopteran parasitoids and predatory species that attack *A. ironsidei*, have been previously reported in macadamia orchards in Hawai’i [22,48,49]. Based on field sampling and molecular assays using species-specific primers to detect the presence of *A. ironsidei* in the gut of some predatory species, Gutierrez-Coarite et al. [49] reported a number of lady beetle species (Coleoptera: Coccinellidae), including *Halmus chalybeus* (Boisduval), *Curinus coeruleus* Mulsant, *Scymnodes lividigaster* (Mulsant), *Rhyzobius forestieri* (Mulsant), *Sticholotis ruficeps* Weise, and *Halmus chalybeus* (Boisduval) as predators of *A. ironsidei* in Hawai’i. In another study, Gutierrez-Coarite et al. [48] reported *Encarsia lounsburyi* (Berlese and Paoli) (Aphelinidae) as an important naturally occurring parasitoid of *A. ironsidei* in Hawai’i, achieving over 73% parasitism in macadamia orchards that are regularly pruned and have wild understory plants. Gutierrez-Coarite et al. [48] underscored that extant natural enemies could contribute to the IPM for *A. ironsidei*. Our results corroborate these reports, as diverse potential hymenopteran parasitoids of *A. ironsidei* were trapped and a few were identified in this study. However, the population of these parasitoids was generally too low throughout the trapping periods, even when the populations of *A. ironsidei* were high at both sites, except *E. lounsburyi* in Paauilo during the December–February trapping period, when over 6000 *E. lounsburyi* wasps were trapped.

The parasitoids trapped showed disparities in terms of the attraction to the four colored traps. For example, myrmarid wasps were captured consistently more in lime green, yellow, and dark green traps than in white traps at both Pahala and Paauilo across both trapping seasons. Trichogrammatidae were seemingly more attracted to lime green and dark traps. However, because the abundance of the different parasitoid species trapped throughout the study period was low, resulting in some inconsistencies in their attraction to the four colors across the two study sites and trapping periods, the effects of color were not adequately reflected in our trapping data. Thus, the results may not be very meaningful, especially in the context of developing and optimizing monitoring tools. However, this may not be the case with *E. lounsburyi* because of the high captures found on yellow, lime green, and dark green cards during the second trapping period in Paauilo. The trapping data from this period clearly showed that yellow, lime green, and dark green were more attractive to *E. lounsburyi* than white, suggesting that dark-colored traps may be more effective in monitoring its population than white traps. Other studies have shown that color could be critical for monitoring parasitoids in cropping systems. Holthouse et al. [37] documented that yellow and blue sticky traps are highly effective in monitoring key parasitoids of *Halyomorpha halys* (Hemiptera: Pentatomidae), such as *Anastatus* spp. Motschulsky (Hymenoptera: Eupelmidae), *Telenomus* spp. Haliday (Hymenoptera: Scelionidae), and *Trissolcus* spp. Ashmead (Hymenoptera: Scelionidae). Nevertheless, we cannot provide assertive conclusions based on our results due to the disparities and low abundance of *E. lounsburyi* during the two trapping seasons in Pahala and the first trapping season in Paauilo, but it is important to note that yellow sticky traps have been reported to be attractive to *E. lounsburyi* in other reports [61].

In summary, although the results on the effects of color in trapping both crawlers and adult males of *A. ironsidei*, as well as associated parasitoids, were not consistent, we found that regardless of the color, sticky cards were effective in trapping male *A. ironsidei* adults and associated parasitoids. Substantially more *A. ironsidei* male adults and associated parasitoids were captured on the sticky cards at both sites and across the two trapping periods than on the double-sided sticky tapes. This was not tested or reported in previous studies [22,23]. Therefore, it could be beneficial for further studies to investigate the utility of sticky cards when developing threshold-based management and evaluating new IPM tools, including semiochemical-dependent approaches for monitoring and management of the invasive *A. ironsidei*.

## Figures and Tables

**Table 1:** Mean ± SEM of the bi-weekly captures on different colored sticky traps deployed in Pahala from September 2022 to November 2022.

Trap Color	*A. ironsidei* Male Adults	*A. ironsidei* Crawlers	*Encarsia* *lounsburyi*	*Encarsia* spp.	*Signiphora* spp.	Myrmaridae	Trichogrammatidae	Aphelinidae	Eulophidae	Other Hymenoptera
Yellow	20.40 ± 4.62	1.50 ± 0.43	0.70 ± 0.24	1.60 ± 0.45 a	1.75 ± 0.39 a	4.30 ± 0.72 a	3.75 ± 0.94 bc	0.70 ± 0.27 ab	1.90 ± 0.44	14.73 ± 1.90 a
Lime Green	17.95 ± 4.18	0.75 ± 0.32	0.70 ± 0.21	0.80 ± 0.32 ab	1.15 ± 0.32 ab	2.75 ± 0.56 a	4.55 ± 0.86 b	1.05 ± 0.32 a	2.35 ± 1.01	11.20 ± 0.64 ab
Dark Green	31.80 ± 11.11	1.25 ± 0.95	0.40 ± 0.20	0.40 ± 0.26 b	1.35 ± 0.35 ab	2.70 ± 0.52 a	13.40 ± 2.80 a	0.20 ± 0.09 b	1.15 ± 0.32	12.33 ± 1.97 ab
White	33.40 ± 15.41	1.30 ± 0.53	0.20 ± 0.09	0.20 ± 0.12 b	0.50 ± 0.17 b	0.35 ± 0.15 b	1.45 ± 0.46 c	0.35 ± 0.13 ab	2.25 ± 0.54	7.20 ± 1.31 b
*χ*2	4.736	0.358	2.535	9.577	10.201	31.685	41.348	11.773	1.615	11.442
*p*	0.1922	0.5499	0.2816	0.0083 *	0.0169 *	<0.0001 *	<0.0001 *	0.0082 *	0.2034	0.0096 *

Means with different letters under each column are significantly different *p* ≤ 0.05 (Tukey’s test). * denotes significance at α = 0.05.

**Table 2:** Mean ± SEM of the bi-weekly captures on different colored sticky traps deployed in Pahala from December 2022 to February 2023.

Trap Color	*A. ironsidei* Male Adults	*A. ironsidei* Crawlers	*Encarsia lounsburyi*	*Encarsia* spp.	*Signiphora* spp.	Myrmaridae	Trichogrammatidae	Aphelinidae	Eulophidae	Other Hymenoptera
Yellow	2.50 ± 0.53	0.00 ± 0.00	0.95 ± 0.27	0.35 ± 0.24 a	1.75 ± 0.36	1.85 ± 0.67 a	1.05 ± 0.43	2.05 ± 0.37 a	4.65 ± 1.17	1.95 ± 0.40
Lime Green	2.25 ± 0.48	0.00 ± 0.00	1.00 ± 0.23	0.00 ± 0.00 b	1.75 ± 0.26	1.80 ± 0.32 a	1.75 ± 0.46	2.75 ± 0.76 a	4.05 ± 0.70	1.25 ± 0.32
Dark Green	1.55 ± 0.39	0.00 ± 0.00	0.50 ± 0.21	0.00 ± 0.00 b	1.00 ± 0.29	0.75 ± 0.24 a	2.40 ± 0.85	1.50 ± 0.40 a	3.15 ± 1.12	1.8 ± 0.39
White	1.15 ± 0.42	0.00 ± 0.00	0.45 ± 0.14	0.00 ± 0.00 b	1.00 ± 0.32	0.05 ± 0.05 b	0.60 ± 0.26	0.40 ± 0.13 b	3.00 ± 0.96	1.60 ± 0.39
*χ*2	3.406	n/a	5.018	207.675	5.372	18.430	7.843	24.176	1.434	0.655
*p*	0.1821	n/a	0.0813	<0.0001 *	0.1465	0.0004 *	0.0494	<0.0001 *	0.4882	0.5822

Means with different letters under each column are significantly different *p* ≤ 0.05 (Tukey’s test). * denotes significance at α = 0.05.

**Table 3:** Mean ± SEM of the bi-weekly captures on different colored sticky traps deployed in Paauilo from September 2022 to November 2022.

Trap Color	*A. ironsidei* Male Adults	*A. ironsidei* Crawlers	*Encarsia lounsburyi*	*Encarsia* spp.	*Signiphora* spp.	Myrmaridae	Trichogrammatidae	Aphelinidae	Eulophidae	Other Hymenoptera
Yellow	61.1 ± 22.85	1.11 ± 3.86 a	2.00 ± 0.82	1.40 ± 0.50	0.85 ± 0.28	1.55 ± 0.44 a	0.80 ± 0.41	0.35 ± 0.17	0.3 ± 0.13	8.5 ± 2.15
Lime Green	34.7 ± 7.08	5.6 ± 1.12 ab	5.45 ± 3.77	3.70 ± 2.98	0.9 ± 0.30	1.15 ± 0.40 a	1.05 ± 0.55	0.2 ± 0.09	0.55 ± 0.21	5.65 ± 1.31
Dark Green	73.85 ± 26.73	3.1 ± 0.84 b	0.75 ± 0.35	0.85 ± 0.51	0.75 ± 0.25	0.75 ± 0.26 a	1 ± 0.37	0.1 ± 0.07	0.4 ± 0.18	4.4 ± 0.87
White	27.95 ± 8.61	12.2 ± 3.97 a	1.15 ± 1.00	0.80 ± 0.53	0.45 ± 0.15	0.00 ± 0.00 b	0.5 ± 0.22	0.25 ± 0.12	0.65 ± 0.24	3.75 ± 1.14
*χ*2	6.889	12.590	6.470	2.836	0.636	1853.557	0.755	n/a	n/a	5.047
*p*	0.0755	0.0018 *	0.0909	0.2422	0.5939	<0.0001 *	0.3848	n/a	n/a	0.0802

Means with different letters under each column are significantly different *p* ≤ 0.05 (Tukey’s test). * denotes significance at α = 0.05.

**Table 4:** Mean ± SEM of the bi-weekly captures on different colored sticky traps deployed in Paauilo from December 2022 to February 2023.

Trap Color	*A. ironsidei* Male Adults	*A. ironsidei* Crawlers	*Encarsia* *lounsburyi*	*Encarsia* spp.	*Signiphora* spp.	Myrmaridae	Trichogrammatidae	Aphelinidae	Eulophidae	Other Hymenoptera
Yellow	27.45 ± 6.62	0.50 ± 0.50 a	89.65 ± 37.42 a	0.00 ± 0.00	0.95 ± 0.26 a	0.35 ± 0.15 ab	3.90 ± 1.65	1.45 ± 0.55	0.45 ± 0.25	2.30 ± 0.78
Lime Green	24.80 ± 4.94	0.50 ± 0.50 a	101.45 ± 35.41 a	0.00 ± 0.00	0.40 ± 0.17 b	0.90 ± 0.32 a	2.15 ± 1.06	1.15 ± 0.38	0.05 ± 0.05	1.25 ± 0.54
Dark Green	19.00 ± 3.89	0.00 ± 0.00 b	103.20 ± 43.74 a	0.00 ± 0.00	0.40 ± 0.27 b	0.25 ± 0.12 b	2.35 ± 0.74	1.20 ± 0.47	0.10 ± 0.10	0.90 ± 0.57
White	14.20 ± 3.15	2.10 ± 1.43 a	17.00 ± 4.75 b	0.00 ± 0.00	0.20 ± 0.12 b	0.05 ± 0.05 b	2.85 ± 1.11	0.30 ± 0.15	0.10 ± 0.10	0.75 ± 0.53
*χ*2	5.539	528.352	25.166	n/a	5.083	11.057	4.210	7.811	3.192	3.322
*p*	0.1363	<0.0001 *	<0.0001 *	n/a	0.0242 *	0.0114 *	0.1219	0.0501	0.074	0.19

Means with different letters under each column are significantly different *p* ≤ 0.05 (Tukey’s test). * denotes significance at α = 0.05.

**Table 5:** Pearson correlation coefficients between the captures of *A. ironsidei* male adults on the different colored sticky cards and *A. ironsidei* crawlers on the double-sided sticky tape. Captures on the double-sided sticky tapes were standardized per 6.4516 cm^2^.

	Pahala		Paauilo	
Trap Color	September–November	December to February	September–November	December to February
Yellow	*r* = −0.0945, *p* = 0.6918	*r* = −0.1794, *p* = 0.4492	*r* = 0.6487, *p* = 0.0020 *	*r* = 0.0026, *p* = 0.9913
Lime Green	*r* = −0.3077, *p* = 0.1870	*r* = 0.4591, *p* = 0.0417 *	*r* = 0.4396, *p* = 0.0524	*r* = 0.4420, *p* = 0.0510
Dark Green	*r* = −0.0909, *p* = 0.7030	*r* = 0.3441, *p* = 0.1374	*r* = 0.2501, *p* = 0.2876	*r* = 0.0447, *p* = 0.8517
White	*r* = 0.4901, *p* = 0.0283 *	*r* = 0.1260, *p* = 0.5964	*r* = 0.9326, *p* < 0.0001 *	*r* = 0.7502, *p* = 0.0001 *

* denotes significance at α = 0.05.

## Data Availability

Data will be made available upon request.

## Supplementary Materials

The following supporting information can be downloaded at: https://www.mdpi.com/article/10.3390/insects16020149/s1, Figure S1: (A) Sticky cards deployed on the lower canopy of a macadamia tree; Table S1: Relative no. of trap captures across four colored sticky traps and double-sided sticky tapes in Pahala from September 2022 to November 2022; Table S2: Relative no. of trap captures across four colored sticky traps and double-sided sticky tapes in Pahala from December 2022 to February 2023; Table S3: Relative no. of trap captures across four colored sticky traps and double-sided sticky tapes in Paaulio from September 2022 to November 2022; Table S4: Relative no. of trap captures across four colored sticky traps and double-sided sticky tapes in Paaulio from December 2022 to February 2023.
