# Supplementary material for: Exploiting Trap Type and Color for Monitoring Macadamia Felted Coccid Acanthococcus ironsidei (Williams) and Associated Parasitic Wasps in Macadamia Orchards in Hawai’i
Source: Insects. 2025 Feb 2;16(2):149. doi: 10.3390/insects16020149 (PMC11856603; doi:10.3390/insects16020149)
Supplement: Supplementary file 1 [file insects-16-00149-s001.zip › insects-3408289-supplementary.pdf]

Table S1. Relative no. of trap captures across four colored sticky traps and double-sided sticky tapes in Pahala from September 2022 to November 2022.

Captured	Sticky Cards		Double-Sided Tape	
	Total Captures	% Total Captures	Total Captures	% Total Captures
Male adult *A. ironsidei*	2071	52.99%	8	0.44%
A. ironsidei Crawlers	96	2.46%	1808	99.45%
E. lounsburyi	40	1.02%	0	0.00%
Encarsia spp.	60	1.54%	0	0.00%
Signiphora	95	2.43%	0	0.00%
Myrmaridae	202	5.17%	0	0.00%
Trichogrammatidae	463	11.85%	0	0.00%
Aphelinidae	46	1.18%	0	0.00%
Eulophidae	153	3.92%	2	0.11%
Other Hymenoptera	682	17.45%	0	0.00%
Total Captures	3908		1818	

Table S2. Relative no. of trap captures across four colored sticky traps and double-sided sticky tapes in Pahala from December 2022 to February 2023.

Captured	Sticky Cards		Double-Sided Tape	
	Total Captures	% Total Captures	Total Captures	% Total Captures
Male adult *A. ironsidei*	149	13.64%	0	0.00%
A. ironsidei Crawlers	0	0.00%	83	98.81%
E. lounsburyi	58	5.31%	0	0.00%
Encarsia spp.	7	0.64%	0	0.00%
Signiphora	110	10.07%	0	0.00%
Myrmaridae	89	8.15%	0	0.00%
Trichogrammatidae	116	10.62%	1	1.19%
Aphelinidae	134	12.27%	0	0.00%
Eulophidae	297	27.20%	0	0.00%
Other Hymenoptera	132	12.09%	0	0.00%
Total Captures	1092		84	

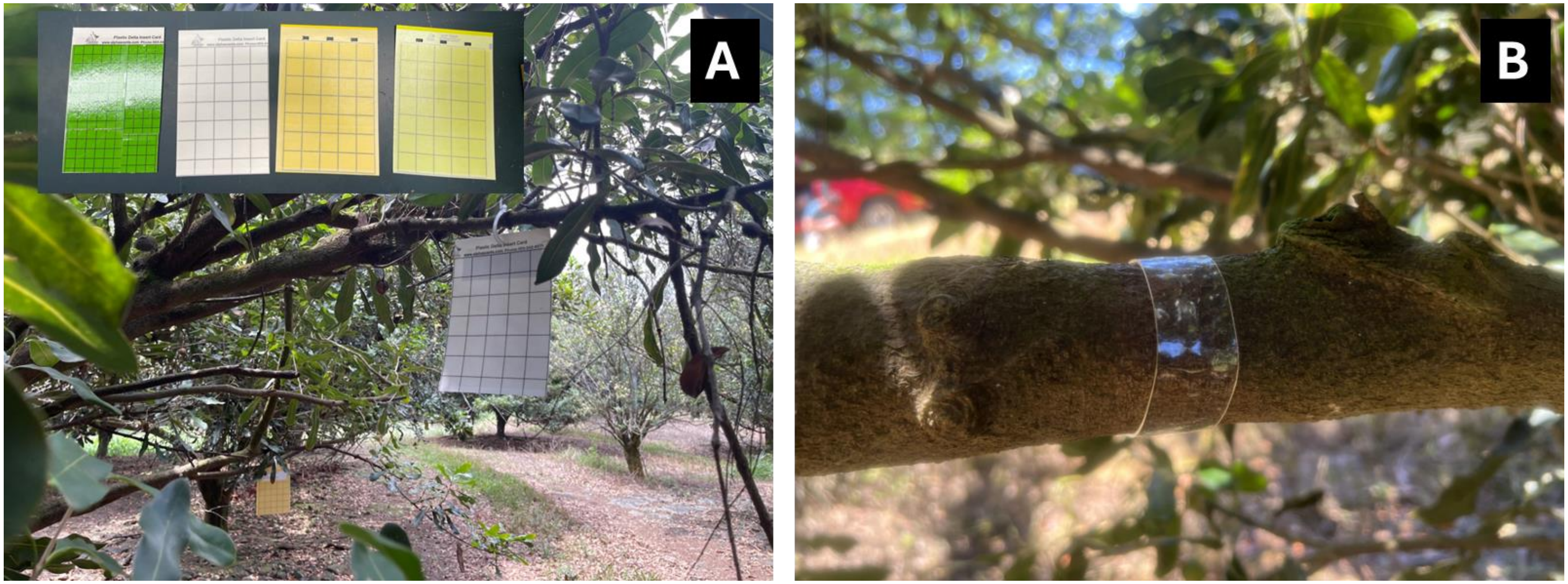

Figure S1. (A) Sticky cards deployed on the lower canopy of a macadamia tree. (A, inset) Different colored sticky cards tested in the study. (B) Double-sided sticky tape wrapped around a macadamia branch.

Table S3. Relative no. of trap captures across four colored sticky traps and double-sided sticky tapes in Paaulio from September 2022 to November 2022.

Captured	Sticky Cards		Double-Sided Tape	
	Total Captures	% Total Captures	Total Captures	% Total Captures
Male adult *A. ironsidei*	3952	70.46%	0	0.00%
A. ironsidei Crawlers	638	11.37%	5063	99.98%
E. lounsburyi	187	3.33%	0	0.00%
Encarsia spp.	135	2.41%	0	0.00%
Signiphora	59	1.05%	0	0.00%
Myrmaridae	69	1.23%	0	0.00%
Trichogrammatidae	67	1.19%	0	0.00%
Aphelinidae	18	0.32%	1	0.02%
Eulophidae	38	0.68%	0	0.00%
Other Hymenoptera	446	7.95%	0	0.00%
Total Captures	5609		5064	

Table S4. Relative no. of trap captures across four colored sticky traps and double-sided sticky tapes in Paaulio from December 2022 to February 2023.

Captured	Sticky Cards		Double-Sided Tape	
	Total Captures	% Total Captures	Total Captures	% Total Captures
Male adult *A. ironsidei*	1709	20.12%	0	0.00%
A. ironsidei Crawlers	62	0.73%	10333	99.95%
E. lounsburyi	6226	73.32%	0	0.00%
Encarsia spp.	0	0.00%	0	0.00%
Signiphora	39	0.46%	0	0.00%
Myrmaridae	31	0.37%	0	0.00%
Trichogrammatidae	225	2.65%	5	0.05%
Aphelinidae	82	0.97%	0	0.00%
Eulophidae	14	0.16%	0	0.00%
Other Hymenoptera	104	1.22%	0	0.00%
Total Captures	8492		10338	
